# Effects of high‐intensity interval training on cardiac function in hypertensive and normotensive men: Effects of antihypertensive treatment

**DOI:** 10.1113/EP093164

**Published:** 2026-01-09

**Authors:** Mads Fischer, Jon Egelund, Matteo Fiorenza, Thomas S. Ehlers, Michael Nyberg, Jesper J. Linde, Lasse Gliemann, Thomas P. Gunnarsson, Jens Bangsbo

**Affiliations:** ^1^ Department of Nutrition, Exercise and Sports University of Copenhagen Copenhagen Denmark; ^2^ Clinical Physiology and Nuclear Medicine Herlev and Gentofte Hospital Herlev Denmark; ^3^ Department of Biomedical Sciences University of Copenhagen Copenhagen Denmark; ^4^ Cardiovascular & Renal Research, Research & Early Development Novo Nordisk A/S Måløv Denmark; ^5^ Department of Cardiology, Rigshospitalet University of Copenhagen Copenhagen Denmark

**Keywords:** blood pressure, cardiac function, echocardiography, hypertension, interval training

## Abstract

Exercise training is recommended for individuals with hypertension because it has been shown to lower blood pressure and reverse left ventricular concentric remodelling and mass. However, it is unclear how hypertensive individuals respond in comparison to normotensive individuals and to what extent medical treatment affects the outcome of training. Our aim was to assess the effect of a 6 week high‐intensity interval training (HIIT) intervention on cardiac adaptations in subjects with treated or untreated essential hypertension compared with normotensive control subjects. Cardiac function was evaluated by echocardiography in 11 medicated hypertensive men, who adhered to treatment during the intervention but refrained from medication for 4 days prior to and during measurements (MED‐HYP), in 9 untreated hypertensive men (HYP) and in 10 age‐matched normotensive men before and after HIIT. At baseline, HYP and MED‐HYP had lower mitral valve *E*/*A* ratio (MED‐HYP, 0.86 ± 0.2; HYP, 0.99 ± 0.1; *P *< 0.05) compared with normotensive men (1.37 ± 0.4). The HIIT stroke volume in normotensive men only improved maximum oxygen uptake (change, 178 ± 239 mL O_2_/min). The *E*/*e*′ ratio (echocardiographic risk marker of cardiac events) increased (*P *< 0.05) with HIIT in MED‐HYP, with no change in normotensive men and HYP. Men with treated and untreated essential hypertension display diminished cardiac adaptation and less improvement in cardiopulmonary fitness in response to HIIT compared with normotensive counterparts. Additionally, MED‐HYP increased *E*/*e*′ with HIIT, potentially raising the risk of primary cardiac events. Therefore, further research is required to assess the interactive effects of exercise training and antihypertensive treatment.

## Abstract (highlights)

- **What is the central question of this study?**
  This study investigates whether hypertension affects exercise‐induced cardiometabolic adaptations and whether the altered blood pressure response seen in individuals on antihypertensive medication reduces these benefits.
- **What is the main finding and its importance?**
  This study reveals that men with essential hypertension (whether treated or untreated) show less improvement in cardiac function and fitness after high‐intensity interval training compared with normotensive peers. Those on antihypertensive medication experienced an increase in a heart failure risk marker (*E*/*e*′ ratio), suggesting that medication might negatively interfere with the potential beneficial cardiac adaptations.

## 1 INTRODUCTION

Cardiovascular disease remains the leading cause of death globally, with essential hypertension being one of the most prevalent risk factors (Andersen et al., 2020; Zhou et al., 2021). Despite antihypertensive pharmacological treatment, many individuals with hypertension continue to display maladaptive cardiac remodelling and an exaggerated blood pressure response to exercise, which elevates their risk of heart failure and mortality (Casale et al., 1986; Chant et al., 2018; Kunutsor et al., 2017). To counteract this unwanted consequences of hypertension, exercise training has been recommended for >50 years (Boyer & Kasch, 1970; Rudd & Day, 1967), because it has several positive health effects, including reduction in blood pressure (Andersen et al., 2020; Cornelissen & Smart, 2013), regression of left ventricular (LV) mass and relative wall thickness (Kamimura et al., 2017; Turner et al., 2000), and reduced risk of cardiovascular adverse events (Devereux, 2004; Whelton, 2002).

The duration, the intensity and the adherence to exercise training are important factors for the antihypertensive effects of exercise training (Costa et al., 2018; Devereux, 2004; Hatle et al., 2014; Wallace, 2003; Whelton, 2002). With time being the most commonly perceived barrier to engage in the recommended amount of exercise (Booth & Laye, 2009; Sweeting et al., 2016), an effective time‐efficient training programme, such as high‐intensity interval training (HIIT), is desirable. Such programmes have also been shown to have a superior effect compared with moderate‐intensity continuous training on maximal oxygen uptake (${\dot{V}}_{O_{2}\max}$) in individuals with hypertension (Costa et al., 2018). Of note, studies have reported significant improvements after a mere 4 weeks of HIIT training in older and populations with cardiac and/or metabolic diseases (Angadi et al., 2015; Heber et al., 2020; Jung et al., 2015; Ross et al., 2016). This is highly desirable, because a higher ${\dot{V}}_{O_{2}\max}$ is linked to a lower risk of coronary heart disease, cardiovascular disease, heart failure and all‐cause mortality (Clausen et al., 2018; Demopoulos et al., 1997; Khan et al., 2014; Kodama, 2009; Laukkanen et al., 2016).

Although both HIIT and antihypertensive medication can lower blood pressure, and HIIT has consistently improved ${\dot{V}}_{O_{2}\max}$, it remains unclear whether HIIT promotes beneficial cardiac adaptations in individuals with hypertension and whether these effects are influenced by concurrent antihypertensive pharmacological treatment. Notably, blood pressure rises with increasing exercise intensity, and individuals with hypertension often exhibit an exaggerated hypertensive (HYP) response during exercise (Delaney et al., 2010). Paradoxically, this malignant blood pressure response has recently been shown to be exaggerated further in individuals receiving antihypertensive treatment compared with individuals who have untreated hypertension or normal blood pressure (Chant et al., 2018). As a result, individuals with hypertension undergoing antihypertensive treatment might be predisposed to blunted exercise‐induced improvements in cardiac and cardiometabolic function.

The aim of the present study was to examine the effect of HIIT on cardiac adaptations in men with treated and untreated essential hypertension and to compare these changes with those in men with normal blood pressure. We hypothesized that HYP individuals undergoing HIIT would exhibit blunted improvements in LV morphology and function compared with normotensive control subjects. Furthermore, we expected that antihypertensive treatment would attenuate these adaptations further, potentially limiting the cardiac benefits of exercise training in treated HYP individuals.

## 2 MATERIALS AND METHODS

### 2.1 Ethical approval

The study was approved by the Ethics Committee of Copenhagen and Frederiksberg communities (H‐4‐2014‐100), as a part of a larger project on HIIT‐induced cardiovascular and muscular changes in individuals with essential hypertension, and was conducted in accordance with the guidelines of the *Declaration of Helsinki*. All participants provided written informed consent prior to enrolment. The study was registered as a clinical trial at ISRCTN.com (ISRCTN11181410). Data on this cohort (24 h blood pressure, muscle sympathetic nerve activity, smooth muscle cell vasodilator function and muscle mitochondrial turnover) have been reported elsewhere (Ehlers et al., 2020; Fiorenza et al., 2019; Gunnarsson et al., 2020b).

### 2.2 Participants

Twenty men with essential hypertension and ten normotensive controls were recruited for the study and divided into three groups: those without hypertension (NORM), those with hypertension not receiving antihypertensive treatment (HYP) and those with hypertension undergoing antihypertensive treatment (MED‐HYP). Recruitment was conducted via public listings. Notably, half of the individuals in the untreated HYP group were unaware of their condition prior to the study, because they were identified through incidental elevated blood pressure measurements. These individuals would otherwise have been classified in the control group. All subjects underwent a medical screening examination by a medical doctor before entering the study. Inclusion criteria were males ≥40 years of age and exercising for <1.5 h per week. Subjects were not included if they were treated with anticoagulants, had a body mass index of >30 kg m^−2^, alcohol intake of >21 units per week, were smokers or had chronic diseases (other than essential hypertension). Throughout the HIIT intervention period, subjects remained medicated with their regular medication, except for the last 4 days leading up to the experimental day (see section 2.4 Experimental days).

### 2.3 Experimental design

The subjects participated in an experimental day before (Pre) and after (Post; within 5 days of the last training session) a 6 week HIIT intervention (see section 2.5 Training intervention).

**FIGURE 1:**
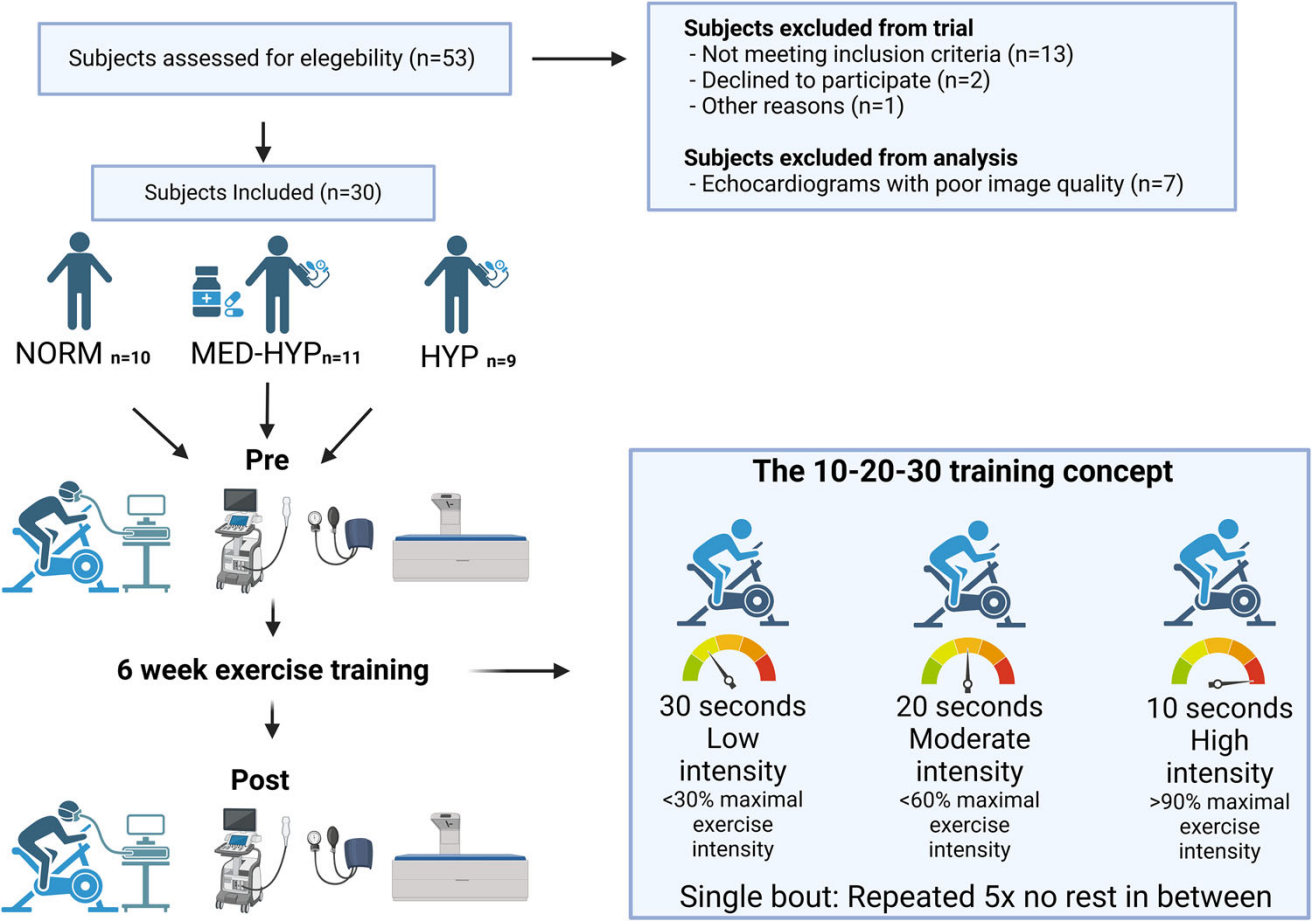
Schematic diagram of the study design. Groups are denoted as normotensive (NORM), untreated hypertensive (HYP) and medicated hypertensive (MED‐HYP). Created in BioRender. Fischer et al. (2025) https://BioRender.com/o90w838.

A schematic diagram of the study design for examining the effect of HIIT on cardiac adaptations is provided in Figure 1.

### 2.4 Experimental days

All subjects were asked to refrain from caffeine, alcohol and exercise for 24 h prior to the experimental days.

On the day of the experiment, after a 20 min period of rest in a supine position in a quiet and dimly lit room, blood pressure was measured. This involved obtaining six consecutive readings using an automatic upper‐arm blood pressure monitor (M7; OMRON, Vernon Hills, IL, USA). The data were analysed and presented as average systolic, diastolic and mean arterial pressure (MAP) (Ehlers et al., 2020).

After the blood pressure measurement, the subject was positioned from the supine position to a left lateral supine position. Transthoracic echocardiography was performed using a GE Vivid E9 ultrasound machine with a 2.5 MHz transducer (GE Healthcare) according to the current guidelines (Zhou et al., 2021). One investigator (J.E.) performed the echocardiographic examinations. To reduce analytical variability, a single investigator (M.F.) analysed all echocardiographic examinations using semi‐automated quantification of two‐dimensional LV volumes and wave tracing. All examinations were analysed offline using EchoPac software v.203 (GE Healthcare) in a random, unidentifiable order, with the investigator blinded to blood pressure characteristics and medicinal status.

The LV mass was calculated according to the Cube formula (Lang et al., 2015) and relative wall thickness was calculated according to Equation 1. Intraventricular septal thickness in diastole (IVSd), LV diameter in diastole (LVIDd; in centimetres) and the posterior wall thickness in diastole (PWTd) were measured. The LV mass was then divided by total body surface area (BSA; Equation 2; DuBois & DuBois, 1915) to have LV mass indexed to BSA (LV mass index BSA), in addition to fat‐free mass (FFM) for LV mass index FFM (in grams per kilogram of FFM) (Bella et al., 1998; DuBois & DuBois, 1915; Lang et al., 2015). All volumetric measurements (LV end‐diastolic, end‐systolic and stroke volume) were evaluated using Simpsons biplane method.

(1) $$\mathrm{Relative}\;\mathrm{wall}\;\mathrm{thickness}=\left(2\times \mathrm{PWTd}\right)/\;\mathrm{LVIDd}$$

(2) $$\mathrm{BSA}\;\left(m^{2}\right)=0.202\times {\left(\mathrm{body}\;\mathrm{mass}\right)}^{0.425}\times {\left(\mathrm{height}\right)}^{0.725}$$

### 2.5 Training intervention

The 6 week training intervention consisted of two weekly sessions in the first 2 weeks, followed by three sessions per week during the final 4 weeks. The training sessions were conducted according to the 10–20–30 training concept, with interval bouts repeated five times (i.e., 5 min), with 3 min of recovery in between sets. The training concepts are illustrated in Figure 1 and described in detail by Gunnarsson and Bangsbo (2012). All training was on a bike ergometer, supervised, and participants wore heart rate monitors (Ehlers et al., 2020; Fiorenza et al., 2019).

### 2.6 Statistical analysis

Our hypotheses were evaluated using a mixed‐effects model framework, with ‘Group’ [normotensive (NORM), MED‐HYP and HYP] and ‘Time’ (Pre and Post training) as fixed factors and with participants as a random factor. *Post hoc* multiple comparisons were corrected using Bonferroni correction, and age and height comparisons between groups were made using Student's unpaired *t*‐tests. Data are reported as the mean ± SD, and statistical analyses were conducted using Prism 9 (version 9.2.0; GraphPad Prism, San Diego, CA, USA), with α set at 0.05 and trends reported for *P*‐values of <0.10. Our primary variable of interest the ratio of the early transmitral inflow velocity (E) to the early diastolic mitral annular tissue velocity (e′), expressed as the ratio (*E*/*e*′) was selected based on its clinical relevance and expected sensitivity to the intervention. Power analysis using G*Power v.3.1.9.7 (Faul et al., 2007) indicated that with a sample size of 10 participants, we would achieve an estimated statistical power of 0.96 for detecting a meaningful effect, assuming typical variance and effect size from prior studies (Schmidt et al., 2013). This exceeds the commonly accepted threshold for sufficient power (≥0.80), suggesting that our study was adequately powered to detect significant changes in *E*/*e*′ (Faul et al., 2007).

## 3 RESULTS

After assessment of eligibility, 9 individuals with untreated hypertension (HYP) and 11 with treated HYP (MED‐HYP) were included in the study (Figure 1). In addition, 10 age‐matched healthy normotensive men (NORM) were included in the study (Figure 1).

The 11 MED‐HYP subjects had one or more of the following medications: angiotensin‐converting enzyme inhibitors (*n* = 4), angiotensin II receptor blockers (*n* = 5), Ca^2+^ antagonist (*n* = 2) and/or diuretics (*n* = 7). Details about medication have been reported previously (Ehlers et al., 2020).

### 3.1 Adherence

Adherence to the 16 training sessions was 15.3 ± 1.1 (95.8% ± 7%) for MED‐HYP, 15.8 ± 0.6 (98.8% ± 4%) for HYP, and 15.8 ± 0.6 (98.8% ± 4%) for normotensive.

### 3.2 Resting heart rate and blood pressure

Heart rate at rest did not differ between groups and was unaffected by HIIT (Table 1). Systolic, diastolic and mean arterial blood pressures were higher (*P* < 0.05) in the HYP and MED‐HYP groups compared with the NORM group, both before and after HIIT (Table 1). Systolic blood pressure (SBP) tended to be reduced after HIIT in the MED‐HYP group (−7.8 ± 8.3 mmHg; *P* = 0.052) but was unchanged in the HYP (−2.2 ± 6.2 mmHg; *P* > 0.05) and NORM groups (−0.5 ± 11.1 mmHg; *P* > 0.05). Diastolic and mean arterial blood pressures did not differ between groups and were unaffected by HIIT.

**TABLE 1:** Subject characteristics and physical performance.

	Normotensive (*n* = 10)		MED‐HYP (*n* = 11)		HYP (*n* = 9)		Fixed effects		
Variable	Pre	Post	Pre	Post	Pre	Post	Time	Group	Time × group
Age, years	57.6 ± 9.3		62.5 ± 5		59.9 ± 7.8				
Height, m	1.82 ± 7		1.77 ± 0.1		1.80 ± 0.1				
Body mass, kg	85.4 ± 13.2	84.3 ± 12.9	82.4 ± 7.0	81.7 ± 7.4	92.3 ± 5.7	91.1 ± 7.0	**0.0024**	0.1055	0.6781
Body surface area, m^2^	2.1 ± 0.2	2.0 ± 0.2	2.0 ± 0.1	2.0 ± 0.1	2.1 ± 0.1	2.1 ± 0.1			
Blood pressure									
Systolic, mmHg	123 ± 13^†^, ^#^	122 ± 11^†^, ^#^	163 ± 18	156 ± 16	151 ± 9	149 ± 11	0.0627	**<0.0001**	0.2496
Diastolic, mmHg	73 ± 6^†^, ^#^	74 ± 9^†^, ^#^	91 ± 5	88 ± 5	89 ± 7	87 ± 8	0.1650	**<0.0001**	0.5945
Mean arterial pressure, mmHg	90 ± 8^†^, ^#^	90 ± 9^†^, ^#^	115 ± 6	111 ± 6	110 ± 7	107 ± 8	0.1070	**<0.0001**	0.4460
Resting HR, beats/min	57 ± 9	55 ± 10	59 ± 5	57 ± 8	63 ± 8	62 ± 10	0.1821	0.2278	0.9925
Body composition									
FFM, kg	57.4 ± 8.9	57.7 ± 8.6	53.6 ± 4	54.2 ± 4.1	58.8 ± 3.3	59.7 ± 3.3	**0.0076**	0.1593	0.5395
Fat mass, kg	25.0 ± 5.4	23.5 ± 5.4^*^	25.7 ± 4.3	24.6 ± 4.7	30.2 ± 4.3	28.1 ± 5^*^	**0.0004**	0.0776	0.2457
Fat percentage, %	30.2 ± 4.4	28.8 ± 4.3^*^	32.3 ± 3.4	30.9 ± 3.7^*^	33.8 ± 3.4	31.8 ± 3.7^*^	**<0.0001**	0.1933	0.6112
Cardiorespiratory fitness and exercise capacity									
${\dot{V}}_{O_{2}\max}$, mL/min	3141 ± 547	3319 ± 647^*^	2723 ± 437	2813 ± 471	3099 ± 478	3130 ± 491	**0.0128**	0.1856	0.2843
${\dot{V}}_{O_{2}\max}$, mL/kg/min	36.8 ± 3.5	39.4 ± 4.5^*^	32.6 ± 4.3	34.0 ± 4.7	33.5 ± 5.4	34.5 ± 5.9	**0.0006**	0.0886	0.2882
Time to exhaustion, s	611 ± 167	699 ± 186^*^	467 ± 96	539 ± 103	554 ± 97	617 ± 99^*^	**0.0002**	0.0688	0.9261
IPPO, W	283.6 ± 55.6^#^	313 ± 61.9^#^, ^*^	218 ± 40	251 ± 45^*^	255 ± 36	286 ± 33^*^	**<0.0001**	**0.0285**	0.9247
IPPO/kg, W/kg	4.9 ± 0.5	5.3 ± 0.4^*^	4.0 ± 0.6	4.6 ± 0.6^*^	4.3 ± 0.5	4.8 ± 0.5^*^	**<0.0001**	**0.0060**	0.7813
IPPO/kg, Watt/kg FFM	3.3 ± 0.5	3.6 ± 0.4	2.6 ± 0.4	3.0 ± 0.5	2.8 ± 0.4	3.2 ± 0.5	**<0.0001**	0.7884	0.9668

*Note*: The table shows characteristics of healthy normotensive, medicated‐hypertensive (MED‐HYP) and hypertensive (HYP) subjects before (Pre) and after (Post) 6 weeks of high‐intensity exercise training. Values are the mean ± SD. Abbreviations: FFM, fat‐free body mass; IPPO, peak power output during the incremental exercise test; ${\dot{V}}_{O_{2}\max}$, maximal pulmonary oxygen consumption.

* Different (*P *< 0.05) from Pre.

^#^ Different (*P *< 0.05) from medicated HYP for the same time point.

^†^ Different (*P *< 0.05) from non‐medicated HYP for the same time point.

### 3.3 Left ventricle mass and volume

**FIGURE 2:**
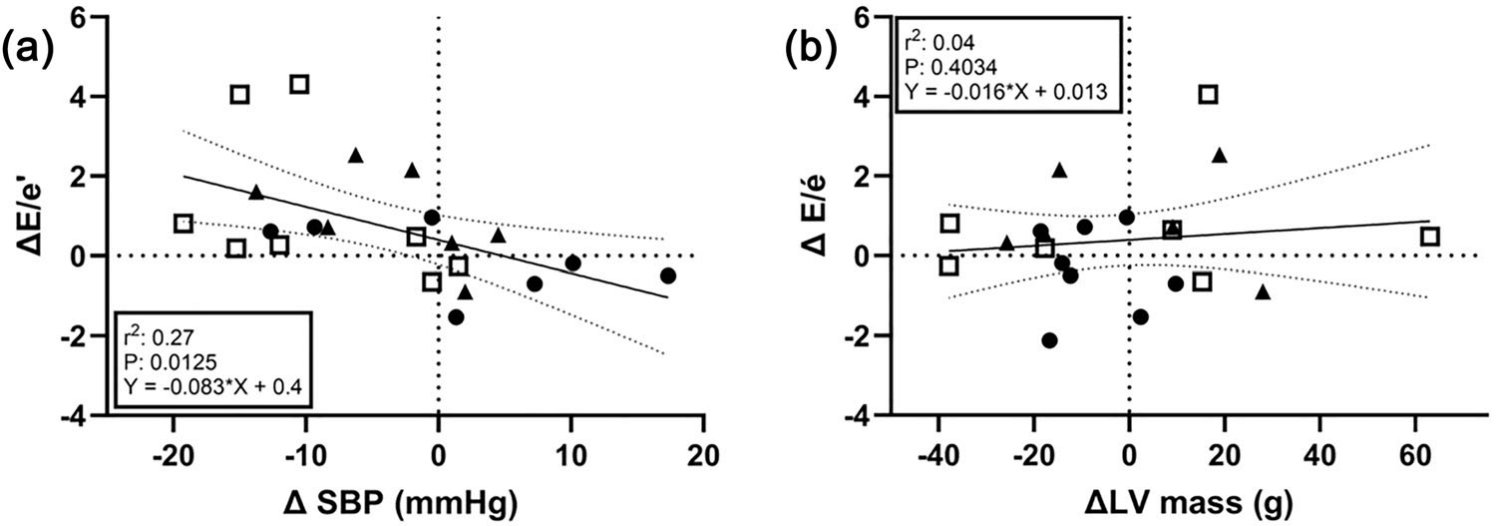
Individual relationship between changes in *E*/*e*′ ratio and changes in: (a) systolic blood pressure (SBP); and (b) left ventricular mass (LV mass). Groups are denoted as normotensive (filled circles), untreated hypertensive (open squares) and medicated hypertensive (filled triangles).

The LV interventricular septal thickness and volumes did not differ between groups and were unaffected by HIIT (*P* > 0.05) in any group (Table 2). The LV mass was also unaffected by HIIT (NORM, −9 ± 12 g; MED‐HYP, −6 ± 24 g; HYP, 0 ± 27 g; all *P* > 0.05) and was not correlated with changes in SBP (Figure 2). A group effect was observed for LV radius‐to‐wall thickness and LV posterior wall thickness (*P* < 0.05), indicating structural differences between groups independent of HIIT. The FFM‐indexed LV mass and absolute LV mass were similar between groups at baseline and were unaffected by HIIT (Table 2). The LV ejection fraction did not differ between groups and was unaffected by HIIT.

**TABLE 2:** Echocardiographic measures before and after 6 weeks of high‐intensity interval training.

	Normotensive (*n* = 10)		MED‐HYP (*n* = 11)		HYP (*n* = 9)		Fixed effects		
Variable	Pre	Post	Pre	Post	Pre	Post	Time	Group	Time × group
Left ventricle mass and volumes									
LVSd, cm	1.3 ± 0.2	1.3 ± 0.2	1.5 ± 0.2	1.5 ± 0.3	1.5 ± 0.3	1.5 ± 0.1	0.9346	0.1332	0.9873
LVIDd, cm	4.8 ± 0.3	4.9 ± 0.3	4.5 ± 0	4.7 ± 0.4	4.9 ± 0	4.9 ± 0.4	0.1164	0.1563	0.3521
LVPWd, cm	1.1 ± 0.1	1.0 ± 0.1^#^	1.2 ± 0.0	1.2 ± 0.2	1.2 ± 0.0	1.1 ± 0.1	0.2033	**0.0364**	0.6865
Radius to wall thickness	0.46 ± 0.05	0.43 ± 0.07	0.54 ± 0.10	0.51 ± 0.09	0.48 ± 0.05	0.47 ± 0.05	0.1221	**0.0372**	0.8568
LV mass, g	229 ± 29	220 ± 32	240 ± 29	248 ± 32	257 ± 32	257 ± 30	0.6844	0.0576	0.5255
LV mass index, g/m^2^	112 ± 7.4	107 ± 9	120 ± 14	124 ± 14	120 ± 13	123 ± 13	0.9178	0.0650	0.5091
LV EDV, mL	126 ± 24	135 ± 25	117 ± 19	118 ± 23	127 ± 15	128 ± 16	0.1653	0.3689	0.2964
LV ESV, mL	52 ± 10	56 ± 11	51 ± 10	54 ± 13	56 ± 8	63 ± 8	0.0558	0.2950	0.9583
LV EF, %	58 ± 5	58 ± 5	57 ± 5	55 ± 4	56 ± 2	51 ± 5	0.0868	0.1060	0.3158
Cardiac output, L/min	4.0 ± 0.6	4.4 ± 0.9	4.2 ± 1	3.9 ± 0.4	4.5 ± 1	4.4 ± 0.6	0.7828	0.4649	0.1445
Left atrial diameter, cm	3.4 ± 0.4	3.5 ± 0.3	3.5 ± 0.3	3.5 ± 0.3	3.6 ± 0.3	3.6 ± 0.3	0.3383	0.5748	0.3158
Left ventricle function									
LVOT VTI	22.8 ± 5	22.1 ± 4	26.2 ± 4	22.1 ± 3^*^	23.5 ± 4	22.5 ± 5	**0.0088**	0.6699	**0.0242**
Mitral valve *E* velocity, cm/s	85 ± 19^#^	77 ± 15	65 ± 10	70 ± 13	72 ± 4	66 ± 9	0.1455	0.0547	**0.0241**
Mitral valve *A* velocity, cm/s	63 ± 12	53 ± 13^#^	80 ± 20	81 ± 16	74 ± 12	64 ± 26	0.1056	**0.0463**	0.1292
Mitral valve *E*/*A* ratio	1.37 ± 0.4^†^, ^#^	1.49 ± 0.3^†^, ^#^	0.86 ± 0.2	0.89 ± 0.2	0.99 ± 0.1	0.92 ± 0.1	0.7713	**<0.0001**	0.5636
Mitral deceleration time, ms	219 ± 29	202 ± 47	243 ± 38	221 ± 28	232 ± 36	204 ± 31	**0.0064**	0.2835	0.9202
PW TDI *s*′, cm/s	12.2 ± 2.6	11.1 ± 1.0	10.6 ± 2.4	9.1 ± 1.6	9.2 ± 0.9	8.9 ± 1.4	0.0925	0.5024	0.2510
PW TDI *e*′, cm/s	12.1 ± 1.6^#^	10.5 ± 1.6^#^	10 ± 2.0	8.6 ± 1.3	10.3 ± 1.7	8.9 ± 1.6	**0.0029**	**0.0059**	0.9053
PW TDI *a*′, cm/s	9.3 ± 1.9	8.9 ± 1.7	13.1 ± 2.1	11.4 ± 2.3	11.9 ± 1.5	11.2 ± 1.6	**0.0076**	0.6136	0.6803
Right ventricular function									
TAPSE, cm	3.1 ± 0.5	3.3 ± 0.3**^†^**	2.7 ± 0.4	2.7 ± 0.3	2.8 ± 0.3	2.4 ± 0.6	0.6049	**0.0202**	0.0642
PW TDI TA *s*′, cm/s	15.8 ± 2.3	16.3 ± 2.9	15.8 ± 3	14.1 ± 3.3	14.7 ± 3	14.3 ± 2.6	0.3852	0.5088	0.2428
PW TDI TA *e*′, cm/s	15.4 ± 4.1	14.9 ± 3.2	13.0 ± 4.0	12.7 ± 3.1	14.0 ± 3.0	14.0 ± 3.8	0.9383	0.7062	0.7761
PW TDI TA *a*′, cm/s	14.1 ± 3.2	15.7 ± 3.0	16.4 ± 3.0	16.4 ± 2.9	14.2 ± 2	13.2 ± 1.8	0.1067	0.1419	0.3022

*Note*: Echocardiographic characteristics of normotensive, medicated hypertensive (MED‐HYP) and non‐medicated hypertensive (HYP) subjects before (Pre) and after (Post) 6 weeks of high‐intensity exercise training (Casale et al., 1986; Chant et al., 2018; Cirener et al., 2024; Clausen et al., 2018; Cornelissen & Smart, 2013; Costa et al., 2018; Delaney et al., 2010; Demopoulos et al., 1997; Devereux, 2004; DuBois & DuBois, 1915; Egelund et al., 2017; Ehlers et al., 2020; Faul et al., 2007; Fiorenza et al., 2019; Fischer et al., 2025; Guirado et al., 2012; Gunnarsson & Bangsbo, 2012; Gunnarsson et al., 2020a, 2020b; Hatle et al., 2014; Heber et al., 2020). Values are the mean ± SD. Abbreviations: EF, ejection fraction; FFM, fat‐free body mass; LV, left ventricular; LVEDV, left ventricular end‐diastolic volume; LVESV, left ventricular end‐systolic volume; LVIDd, left ventricular end‐diastolic diameter; LVOT VTI, left ventricular outflow tract velocity–time integral; LVPWd, left ventricular posterior wall thickness at diastole; LVSd, left ventricular interventricular septum at diastole; PW TDI, pulsed wave tissue Doppler imaging; TA, tricuspid annular; TAPSE, tricuspid annular plane systolic excursion.

* Different (*P *< 0.05) from Pre.

^#^ Different (*P *< 0.05) from MED‐HYP for the same time point.

^†^ Different (*P *< 0.05) from HYP for the same time point.

### 3.4 Left ventricular function

**FIGURE 3:**
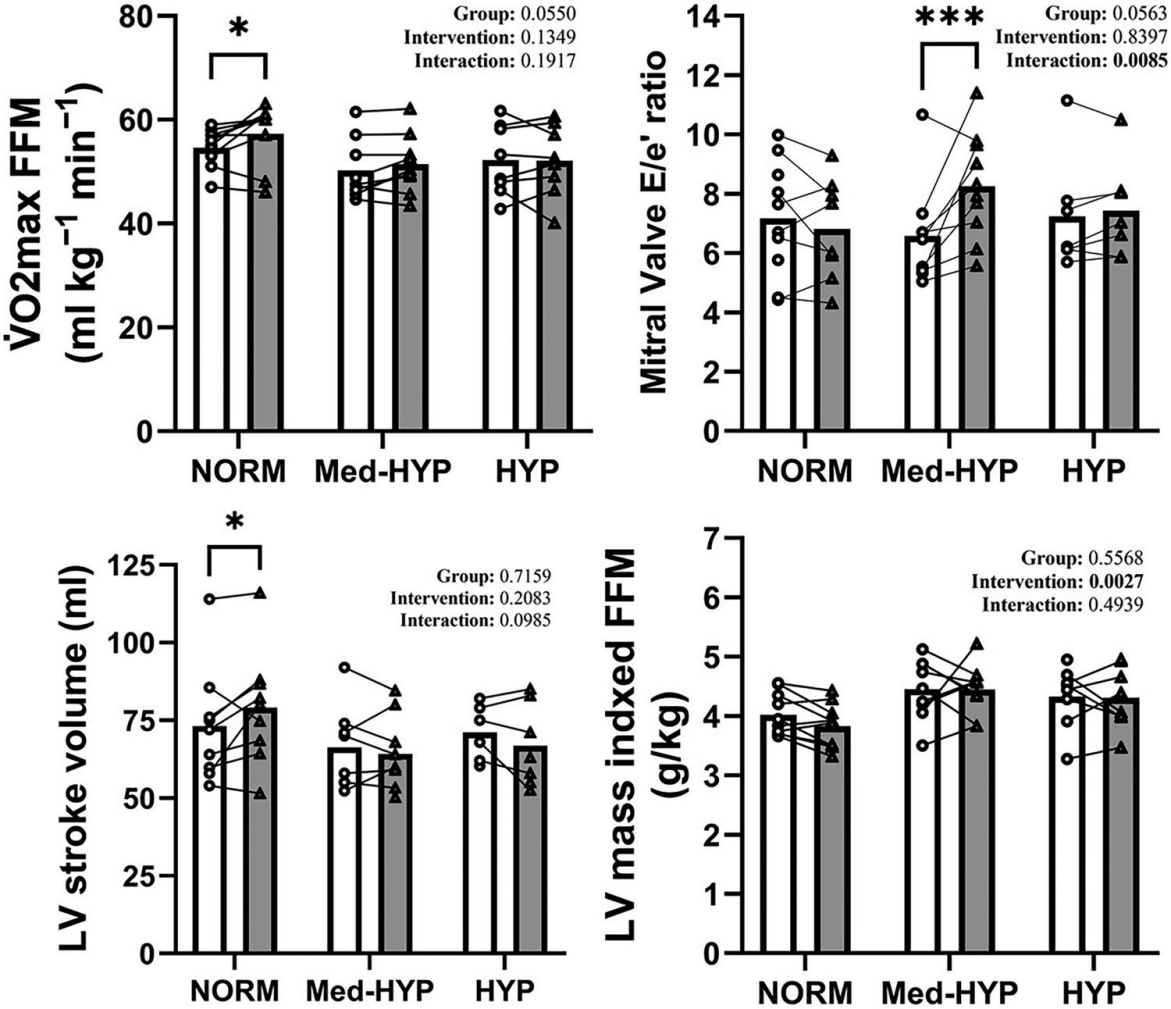
Maximal oxygen uptake (${\dot{V}}_{O_{2}\max}$) expressed per fat‐free mass (FFM), left ventricular (LV) mass expressed per FFM, mitral valve *E*/*e*′ ratio and LV stroke volume (in millilitres) before (PRE; white bars and open circles), and after (POST; grey bars and filled triangles) the training intervention. Groups: HYP, untreated hypertension; NORM, normotensive; and MED‐HYP, medicated hypertensive. ^*^ *P *< 0.05; ^***^ *P *< 0.001.

A time × group interaction was observed for the *E*/*e*′ ratio, mitral valve (MV) early inflow velocity and LV outflow tract velocity–time integral (Table 2 and Figure 3). The *E*/*e*′ ratio increased with HIIT in MED‐HYP (1.7 ± 1.7; *P* < 0.05) but was unchanged in the NORM (−0.3 ± 1.1; *P* > 0.05) and HYP groups (−0.7 ± 2.7; *P* > 0.05). The MV early inflow velocity did not differ between groups and was unaffected by HIIT. The LV outflow tract velocity–time integral decreased with HIIT in MED‐HYP (−3.7 ± 2.8 cm; *P* < 0.05) but was unchanged in NORM and HYP groups. The MV early‐to‐late filling ratio (MV *E*/*A*) was higher (*P* < 0.05) in the NORM group compared with both Med‐HYP and HYP at baseline and remained higher after HIIT. Changes in the *E*/*e*′ ratio were negatively correlated with the changes in SBP (Figure 3). Of the two measures in the *E*/*e*′ ratio, changes in MV early inflow velocity were the cause of the association between the ratio of mitral inflow velocity to early diastolic myocardial tissue velocity (*e*′) and SBP (ΔMV *E* vs. ΔSBP; *R* ^2^ = 0.27; see Figure 3). In contrast, *e*′ was not correlated with changes in SBP (Δ*e*′ vs. ΔSBP; *R* ^2^ = 0.03). Peak systolic mitral annular systolic velocity (*S*′) did not differ between groups and was unaffected by HIIT.

### 3.5 Right ventricular function

No time × group interaction was found in any of the right ventricular functional measures, and no differences were found within any of the groups with HIIT (Table 2).

### 3.6 Body composition

No significant time × group interaction was found in any of the measures of body composition (Table 1). Body mass, FFM and BSA did not differ between groups and were unaffected by HIIT at the group level (Table 1). Body mass decreased overall with HIIT (−1.0 ± 1.6 kg) but did not change significantly within any of the groups (MED‐HYP, −0.63 ± 1.2 kg; HYP, −1.2 ± 2.1 kg; NORM, −1.1 ± 1.5 kg; all *P* > 0.05). The FFM increased overall with HIIT (0.6 ± 1.1 kg) but did not change significantly within any of the groups (MED‐HYP, 0.6 ± 1.0 kg; HYP, 0.9 ± 1.4 kg; NORM, 0.3 ± 1.1 kg; all *P* > 0.05). Fat percentage decreased with HIIT in all groups (MED‐HYP, −1.4% ± 1.3%; HYP, −2.0% ± 2.0%; NORM, −1.4% ± 1.4%; all *P* < 0.05).

### 3.7 Maximum oxygen uptake and exercise capacity

The ${\dot{V}}_{O_{2}\max}$ and time to exhaustion during the incremental test increased with HIIT only in the NORM group (${\dot{V}}_{O_{2}\max}$, 178 ± 239 mL/min; time to exhaustion, 74.5 ± 42.9 s; both *P* < 0.05), with no changes observed in MED‐HYP or HYP (Table 1). Peak power output increased with HIIT in all groups (MED‐HYP, 33 ± 26 W; HYP, 31 ± 21 W; NORM, 29 ± 17 W; all *P* > 0.05), with no difference between groups (Table 1).

## 4 DISCUSSION

The main findings of this study were that HIIT‐induced adaptations in stroke volume and ${\dot{V}}_{O_{2}\max}$ were observed only in the normotensive subjects and were not apparent in the hypertensive subjects (Table 1 and supplemental table 1 and 2). In addition, the exercise training intervention increased *E*/*e*′, a predictor of cardiac adverse events in HYP populations (Sharp et al., 2010; Zhou et al., 2018), only in the MED‐HYP individuals but not in those with untreated hypertension or normal blood pressure (Fig. 2).

The exercise training intervention resulted in an increased *E*/*e*′ ratio in MED‐HYP exclusively and could suggest that HIIT has deleterious effects on diastolic function in individuals receiving antihypertensive treatment (Biagini et al., 2009; Sharp et al., 2010; Zhou et al., 2018). The influence of exercise training on cardiac function in medically treated individuals with hypertension has been inadequately explored, and to date, no other study on cardiac adaptations in individuals with hypertension includes a normotensive or untreated HYP control group. To our knowledge, only two studies have examined the effects of training on cardiac diastolic function in treated HYP individuals (Guirado et al., 2012; Kelemen et al., 1990), and only the most recent included estimates of diastolic function and filling pressure (i.e., *E*/*e*′). That study used a 6 month supervised exercise programme with three weekly sessions of combined aerobic and resistance training, reporting vigorous aerobic intensity but without specifying duration, and strength training without noting targeted muscle groups; although it improved cardiorespiratory fitness and reduced blood pressure, it did not affect cardiac morphology (Guirado et al., 2012). The cause of the discrepancy in the cardiac adaptations between that study and our study is unclear but might be attributed to the training intensity and/or modality used in each study. This discrepancy might be attributable to the difference in training duration, volume of high‐intensity exercise and/or the inclusion of resistance training, all of which have the potential to impact the cardiac remodelling and function (Arbab‐Zadeh et al., 2014; Levine et al., 2015; Turner et al., 2000).

During normal ageing, both the MV *E* and *e*′ decline progressively (Cirener et al., 2024; Nio et al., 2015). However, the decline in *e*′ is proportionally greater, leading to an age‐related increase in the ratio between mitral inflow velocity and *e*′ (Nio et al., 2015; Robinson et al., 2024). Both parameters are load dependent and typically decrease with increasing afterload, which might account, in part, for the observed association between the *E*/*e*′ ratio and SBP (Figure 2). Although this raises the question of whether the health benefits of lowering SBP might outweigh the *E*/*e*′ estimated cardiac risk, findings from the Anglo‐Scandinavian Cardiac Outcomes Trial (ASCOT) indicate that *E*/*e*′ is an independent risk factor (Sharp et al., 2010). Specifically, the association between *E*/*e*′ and adverse outcomes remained significant even after adjustment for diabetes and SBP (Sharp et al., 2010). The consistent decline in *e*′ across all groups was unexpected but aligned with findings from studies involving strength training (Baggish et al., 2008; Schmidt et al., 2014). In contrast, studies implementing endurance training typically report increases in *e*′ (Baggish et al., 2008; Schmidt et al., 2014). One possible explanation is that, across all groups, average LV mass and heart rate showed small, non‐significant decreases. Given the established positive association between *e*′ and both LV mass (Baggish et al., 2008) and heart rate (Fischer et al., 2025; Quintana et al., 2004), these decreases might have contributed to the observed reduction in *e*′. Most studies in healthy subjects involving prolonged exercise interventions report that increases in LV mass and only minor reductions in heart rate (Arbab‐Zadeh et al., 2014; Baggish et al., 2008) lead to increases in *e*′, which was not observed in our study.

Only the normotensive group experienced an improvement in ${\dot{V}}_{O_{2}\max}$ after the training intervention. Both NORM and HYP groups showed increased time to exhaustion, whereas the MED‐HYP group exhibited no improvements in either measure. Despite varying improvements in ${\dot{V}}_{O_{2}\max}$, all three groups improved peak power performance in the incremental exercise test, which might be indicative of improved ion handling and fatigue resistance (Hostrup & Bangsbo, 2017). These findings suggest that the normotensive individuals had improvements in both central and peripheral adaptations, whereas the HYP groups had mainly peripheral adaptations. The absence of cardiometabolic fitness gains in the HYP groups might relate, in part, to the relatively short intervention duration (6 weeks) and the very brief lead‐in period (1 week) before the full HIIT programme was initiated. Notably, longer interventions with progressive increments in exercise load have been shown to reverse LV chamber and myocardial stiffening in patients with LV hypertrophy and heart failure with preserved ejection fraction (Hieda et al., 2021).

Several studies assessing the potential effects of medication and exercise adaptations have found no interaction on blood pressure and gross cardiac morphological measures (wall thickness and ventricular diameters) with the use of antihypertensive medications, such as diltiazem (calcium channel blocker), metoprolol (β_1_‐adrenergic receptor blocker) and propranolol, with training (Kelemen et al., 1990; Stewart et al., 1990; Wallace, 2003). However, propranolol (β_1_‐ and β_2_‐adrenergic receptor blocker) has been shown to impair ${\dot{V}}_{O_{2}\max}$ after an acute dose and after chronic use following a training period (Kelemen et al., 1990; Stewart et al., 1990). These findings raise questions about the optimal balance between exercise prescription (frequency, duration and intensity) and pharmacological treatment. In addition, given that all participants in the present study were unmedicated during laboratory visits, it remains uncertain to what extent the medication they were prescribed might have influenced their blood pressure or cardiac function whilst exercising and for MED‐HYP adhering to treatment.

Chant et al. (2018) observed that the SBP response to an acute bout of exercise was similar regardless of medication status in HYP individuals, whereas the diastolic pressure response at exercise intensities >50% of peak oxygen uptake was significantly lower in HYP men not receiving treatment compared with those taking antihypertensives (similar to the treatment used in the present study). This lower diastolic pressure response and the subsequently reduced MAP during exercise in the untreated HYP group might have implications on cardiac work, influencing both acute and long‐term cardiovascular health and exercise tolerance. This blood pressure response ultimately leads to a higher pulse pressure, which might be one of the key factors contributing to the improvements in exercise capacity (time to exhaustion) seen in the unmedicated HYP group, potentially mediated by an exercise‐induced improvement in endothelium‐dependent vasodilator responsiveness, as previously shown in HYP men (Gunnarsson et al., 2020a).

Both the MED‐HYP and HYP groups showed significantly higher LV mass at baseline compared with the NORM individuals, and this did not change with the exercise training intervention. LV hypertrophy is considered an independent risk factor, because patients with LV hypertrophy show higher morbidity and mortality than blood pressure‐matched patients without LV hypertrophy (Casale et al., 1986; Levy et al., 1990). Although exercise training‐induced regression in LV concentric remodelling has been shown to be significantly reduced with longer (6 month) training regimens (Turner et al., 2000) and with an active lifestyle (Palatini et al., 2008), no significant changes in LV concentric remodelling were observed in the present study. This is likely to be attributable to the short‐term duration of the exercise training intervention period.

Unlike studies in strength and endurance athletes, showing that increases in ventricular mass are related to attenuated LV compliance (Baggish et al., 2008), the change in *E*/*e*′ ratio in the present study was not correlated with changes in LV mass (Figure 2b). In both the medicated and non‐medicated HYP groups, 6 weeks of HIIT reduced SBP non‐significantly, by ∼8 and ∼2 mmHg, respectively, and an individual relationship between the increase in *E*/*e*′ and reduced blood pressure was found (Figure 2a). Our present data, therefore, indicate that although HIIT might positively influence SBP it might also elicit reduced diastolic function in HYP individuals, potentially complicating the management of hypertension. The lack of significant reductions in blood pressure is in agreement with findings in other studies using HIIT for a comparable time span (Costa et al., 2018). It is conceivable that a longer training period would have elicited greater blood pressure reductions in addition to higher ${\dot{V}}_{O_{2}\max}$, although ${\dot{V}}_{O_{2}\max}$ has been shown to increase significantly in the initial (4–12 week) phase of an exercise training regimen (Arbab‐Zadeh et al., 2014; Hatle et al., 2014).

In summary, subjects with essential hypertension, regardless of treatment status, showed blunted HIIT‐induced adaptations in cardiac function and cardiorespiratory fitness compared with normotensive subjects. In addition, subjects with hypertension receiving medical treatment displayed a training‐induced increase in *E*/*e*′ ratio, which is associated with increased risk for cardiac events. These results suggest that although the benefit of exercise on blood pressure is clear in individuals with hypertension, hypertension appears to be associated with an overall decrease in exercise responsiveness, and antihypertensive medication might interfere with cardiac adaptations, leading to an increase in risk markers of cardiac events.

### 4.1 Limitations

Given the small sample size and the variability in the measurements, some of the analyses might be underpowered to detect between‐group differences. However, similar sample sizes have previously been sufficient to demonstrate significant interactions in a diabetic population undergoing exercise training, particularly in ${\dot{V}}_{O_{2}\max}$, *E*/*e*′ ratio and LV end‐diastolic volume (Egelund et al., 2017; Schmidt et al., 2013). Additional limitations of the study include the exclusion of women. Future research should incorporate both men and women, assess the effectiveness of various exercise modalities on vascular outcomes in treated and untreated HYP individuals, and implement a long‐term training period.

## 5 CONCLUSION

To our knowledge, this study is the first to examine cardiac adaptations to exercise training in treated and untreated HYP individuals, in comparison to a normotensive control group. Although exercise is widely acknowledged as a cornerstone in hypertension management (Cornelissen & Smart, 2013), our study highlights the need for a tailored approach, especially for those concurrently undergoing antihypertensive medication. In future studies, we recommend the inclusion of a normotensive healthy control group, a female group, groups receiving drug‐type‐specific treatment and incorporaation of individuals who exhibit apparent hypertension at the time of inclusion (i.e., not receiving antihypertensive treatment). This approach would provide additional insights into the altered exercise responsiveness observed in individuals with essential hypertension and the distinct effects of various treatment approaches.

## AUTHOR CONTRIBUTIONS

Thomas P. Gunnarsson and Jens Bangsbo conceived and designed the experimental protocol. Thomas P. Gunnarsson, Matteo Fiorenza, Thomas S. Ehlers, Michael Nyberg and Jon Egelund were responsible for data collection. Data analysis was performed by Mads Fischer and interpretation of the results by Mads Fischer, Lasse Gliemann, Thomas P. Gunnarsson, Jesper J. Linde and Jens Bangsbo. Mads Fischer drafted the manuscript. All authors critically revised the manuscript, approved its final version and agree to be accountable for all aspects of the work in ensuring that questions related to the accuracy or integrity of any part of the work are appropriately investigated and resolved. All persons designated as authors qualify for authorship, and all those who qualify for authorship are listed.

## CONFLICT OF INTEREST

The authors have no conflicts of interest to disclose.

## Supporting information



Supporting Information

Supporting Information

## Data Availability

Deidentified individual participant data that support the findings of this study will be made available from the corresponding author upon reasonable request, in accordance with institutional and ethical approvals.
