# Supplementary material for: Effects of high‐intensity interval training on cardiac function in hypertensive and normotensive men: Effects of antihypertensive treatment
Source: Exp Physiol. 2026 Jan 9;111(6):2945–55. doi: 10.1113/EP093164 (PMC13238882; doi:10.1113/EP093164)
Supplement: Supplementary file 1 — Supporting Information [file EPH-111-2945-s002.docx]

**Supplemental table 1 - Subject characteristics and physical performance**

|  | **NORMOTENSIVE (n = 10)** | | **HYPERTENSIVE (n =20 )** | | **Fixed effects** | | |
| --- | --- | --- | --- | --- | --- | --- | --- |
| **Variable** | **Pre** | **Post** | **Pre** | **Post** | **Time** | **Group** | **Interaction** |
| Age (y) | 57.6 ± 9.3 | | 61.4 ± 6.6 | |  | 0.226 |  |
| Height (cm) | 182 ± 7 | | 177 ± 6 | |  | 0.065 |  |
| Body mass (kg) | 85.4 ± 13.2 | 84.3 ± 12.9 | 86.8 ± 8.1 | 85.9 ± 8.6* | **0.003** | 0.708 | 0.713 |
| **24‐h ambulatory blood pressure** | | | | |  |  |  |
| Systolic (mm Hg) | 123 ± 13 | 122 ± 11 | 157 ± 16**†** | 153 ± 15**†** | 0.154 | **<0.001** | 0.242 |
| Diastolic (mm Hg) | 73 ± 6 | 74 ± 9 | 90.2 ± 6**†** | 87 ± 7**†** | 0.314 | **<0.001** | 0.307 |
| Mean (mm Hg) | 90 ± 8 | 90 ± 9 | 113 ± 7**†** | 109 ± 8**†** | 0.234 | **<0.001** | 0.255 |
| Resting HR (bpm) | 57 ± 9 | 55 ± 10 | 61 ± 7 | 59 ± 9 | 0.199 | 0.219 | 0.985 |
|  |  |  |  |  |  |  |  |
| **Body composition** | | | | |  |  |  |
| Fat free mass (kg) | 57.4 ± 8.9 | 57.7 ± 8.6 | 56 ± 4.5 | 56.7 ± 4.6* | **0.024** | 0.641 | 0.318 |
| Fat mass (kg) | 25.0 ± 5.4 | 23.5 ± 5.4* | 27.7 ± 4.8 | 26.1 ± 5.1* | **<0.001** | 0.192 | 0.774 |
| Fat percentage (%) | 30.2 ± 4.4 | 28.8 ± 4.3* | 33.0 ± 3.5 | 31.3 ± 3.7* | **<0.001** | 0.088 | 0.709 |
|  |  |  |  |  |  |  |  |
| **Cardiorespiratory fitness and exercise capacity** | | | | |  |  |  |
| *V̇*_O2max_ (ml/min) | 3141 ± 547 | 3319 ± 647* | 2900 ± 493 | 2962 ± 506 | **0.004** | 0.186 | 0.141 |
| *V̇*_O2max_ (ml/kg/min) | 36.8 ± 3.5 | 39.4 ± 4.5* | 33 ± 4.9 | 34.2 ± 5.3***†** | **<0.001** | **0.028** | 0.121 |
| Time to exhaustion (sec) | 611 ± 167 | 699 ± 186* | 508 ± 106 | 576 ± 109* | **<0.001** | 0.052 | 0.285 |
| IPPO (Watt) | 283.6 ± 55.6 | 313 ± 61.9* | 235.1 ± 42.3**†** | 267.1 ± 43.4* | **<0.001** | **0.025** | 0.723 |
| IPPO/kg (Watt/kg) | 3.3 ± 0.5 | 3.6 ± 0.4* | 2.7 ± 0.4**†** | 3.1 ± 0.5***†** | **<0.001** | **0.003** | 0.669 |
| IPPO/kg (Watt/kg FFM) | 4.9 ± 0.5 | 5.3 ± 0.4* | 4.2 ± 0.6***†** | 4.7 ± 0.6***†** | **0. 002** | **<0.001** | 0.620 |

Characteristics of healthy normotensive, medicated-hypertensive and hypertensive (HYP) subjects before (Pre) and after (Post) 6 weeks of high‐intensity exercise training. *V̇*_O2_. Pulmonary oxygen consumption; *V̇*_O2max_. Maximal pulmonary oxygen consumption; FFM, fat-free body mass; IPPO. Peak power output during the incremental exercise test. Values are mean ± SD.

^*^Different (*P* < 0.05) from Pre. **^†^**Different (*P* < 0.05) from normotensive.
