# Supplementary material for: Effects of high‐intensity interval training on cardiac function in hypertensive and normotensive men: Effects of antihypertensive treatment
Source: Exp Physiol. 2026 Jan 9;111(6):2945–55. doi: 10.1113/EP093164 (PMC13238882; doi:10.1113/EP093164)
Supplement: Supplementary file 2 — Supporting Information [file EPH-111-2945-s001.docx]

**Supplemental table 2 - Echocardiographic measures before and after 6 weeks of high‐intensity (10-20-30) interval training**

|  | **NORMOTENSIVE (n = 10)** | | **HYPERTENSIVE (n =20 )** | | **Fixed effects** | | |
| --- | --- | --- | --- | --- | --- | --- | --- |
|  | **Pre** | **Post** | **Pre** | **Post** | **Time** | **Group** | **Interaction** |
| **LV mass and volumes** | | | | |  |  |  |
| LVSd (cm) | 1.3 ± 0.2 | 1.3 ± 0.2 | 1.4 ± 0.2 | 1.5 ± 0.2 | 0.845 | 0.063 | 0.742 |
| LVIDd (cm) | 4.8 ± 0.3 | 4.9 ± 0.3 | 4.7 ± 0.5 | 4.8 ± 0.4 | 0.150 | 0.342 | 0.903 |
| LVPWd (cm) | 1.1 ± 0.1 | 1.0 ± 0.1 | 1.2 ± 0.1 | 1.2 ± 0.1 | 0.119 | **0.036** | 0.512 |
| Relative wall thickness | 0.46 ± 0.05 | 0.43 ± 0.07 | 0.52 ± 0.09 | 0.49 ± 0.08 | 0.052 | 0.078 | 0.835 |
| LV mass (g) | 229.2 ± 28.8 | 219.8 ± 30.7 | 241.9 ± 27.7 | 248.6 ± 31.9 | 0.722 | **0.012** | 0.116 |
| LV mass index (g/m^2^) | 111.7 ± 7.4 | 107 ± 8.7 | 118.9 ± 13.3 | 122.8 ± 13.6**†** | 0.722 | **0.012** | 0.116 |
| LV EDV (ml) | 125.5 ± 23.7 | 135.3 ± 24.5 | 121.5 ± 17.6 | 121.8 ± 21.5 | 0.052 | 0.274 | 0.119 |
| LV ESV (ml) | 52.3 ± 10.2 | 56.2 ± 11.2 | 53.7 ± 10 | 56.3 ± 11.6 | 0.060 | 0.974 | 0.797 |
| LV stroke volume (ml) | 73.2 ± 17.3 | 79.1 ± 17.1 | 67.7 ± 10.9 | 65.5 ± 11.9 | 0.215 | 0.093 | **0.032** |
| LV EF (%) | 57.9 ± 5.2 | 58.1 ± 5.2 | 55.9 ± 4.4 | 53.8 ± 4.5 | 0.311 | 0.112 | 0.218 |
| Cardiac Output (L/min) | 4.0 ± 0.6 | 4.4 ± 0.9 | 4.3 ± 0.7 | 4.1 ± 0.6 | 0.376 | 0.970 | **0.041** |
| Cardiac Output (mL/kg FFM) | 67.8 ± 8.6 | 74.3 ± 13.3 | 76.8 ± 12.2 | 73.2 ± 10.4 | 0.533 | 0.420 | **0.044** |
|  |  |  |  |  |  |  |  |
| **Left ventricle function** | | | | |  |  |  |
| LVOT VTI | 22.8 ± 5 | 22.1 ± 4 | 24.8 ± 4 | 22.3 ± 4 | **0.013** | 0.566 | 0.142 |
| Mitral valve E velocity (cm/s) | 84.8 ± 18.7 | 76.9 ± 14.9 | 68.2 ± 8.6**†** | 68.3 ± 11.4 | 0.121 | **0.013** | 0.557 |
| Mitral valve A velocity (cm/s) | 63.4 ± 11.8 | 53 ± 12.6 | 73.3 ± 24 | 73.6 ± 22.8 | 0.067 | 0.341 | 0.367 |
| Mitral valve E/A ratio | 1.37 ± 0.4 | 1.49 ± 0.3 | 0.93 ± 0.2**†** | 0.88 ± 0.2**†** | 0.574 | **<0.001** | 0.247 |
| Mitral deceleration time (ms) | 219 ± 29 | 202 ± 47 | 238 ± 37.3 | 214 ± 30* | **0.010** | 0.186 | 0.790 |
| PW TDI E’ (cm/s) | 12.2 ± 2.6 | 11.1 ± 1.0 | 9.8 ± 1.7**†** | 8.8 ± 1.5***†** | **0.006** | **0.001** | 0.973 |
| PW TDI A’ (cm/s) | 12.1 ± 1.6 | 10.5 ± 1.6 | 12.2 ± 2.6 | 11.6 ± 1.9 | **0.004** | 0.263 | 0.593 |
| PW TDI S’ (cm/s) | 9.3 ± 1.9 | 8.9 ± 1.7 | 9.7 ± 1.9 | 9.2 ± 1.5***†** | 0.208 | 0.663 | 0.758 |
| E/e’ ratio | 7.1 ± 1.9 | 6.8 ± 1.6 | 7.0 ± 1.6 | 7.8 ± 1.7 | 0.454 | 0.586 | **0.031** |
| Global longitudinal strain (%) | -18.5 ± 2.3 | -17.5 ± 2.3 | -18.2 ± 1.9 | -17.6 ± 1.8 | 0.069 | 0.903 | 0.688 |
|  |  |  |  |  |  |  |  |
| **Right ventricle function** | | | | |  |  |  |
| TAPSE (cm) | 3.1 ± 0.5 | 3.3 ± 0.3 | 2.7 ± 0.4 | 2.6 ± 0.5**†** | 0.734 | **0.001** | 0.098 |
| PW TDI TA S’ (cm/s) | 15.6 ± 2.19 | 16.4 ± 3.02 | 15.1 ± 2.7 | 13.8 ± 2.3***†** | 0.575 | 0.140 | **0.011** |
| PW TDI TA E’ (cm/s) | 12.1 ± 1.5 | 14.7 ± 2.8 | 12.1 ± 1.4 | 11.8 ± 1.6**†** | 0.162 | **0.029** | 0.079 |
| PW TDI TA A’ (cm/s) | 14.3 ± 2.1 | 16.9 ± 2.2 | 16.2 ± 3.8 | 15.6 ± 3.3 | 0.342 | 0.520 | **0.013** |

Echocardiographic characteristics of healthy normotensive, medicated-hypertensive and hypertensive subjects before (Pre) and after (Post) 6 weeks of high‐intensity exercise training. FFM. fat-free body mass; EF indicates ejection fraction; IVSd. left ventricular interventricular septum diastole; LVIDd. left ventricular end-diastolic diameter; LVPWd. left ventricular posterior wall thickness diastole; LV. left ventricular; LVEDV. left ventricular end-diastolic volume; LVESV. left ventricular end-systolic volume; TAPSE. tricuspid annular plane systolic excursion; LVOT VTI. Left ventricular outflow tract velocity time integral; PW TDI. Pulsed wave tissue doppler imaging; Tricuspid annular. TA. Values are mean ± SD.

^*^Different (*P* < 0.05) from Pre. **^†^**Different (*P* < 0.05) from hypertensive for the same time-point.
